# Quantitative tools for analyzing rhizosphere pH dynamics: localized and integrated approaches

**DOI:** 10.1093/biomethods/bpaf026

**Published:** 2025-04-03

**Authors:** Poonam Kanwar, Stan Altmeisch, Petra Bauer

**Affiliations:** Institute of Botany, Heinrich Heine University, Universitätsstr. 1, D-40225, Düsseldorf, Germany; Cluster of Excellence on Plant Science (CEPLAS), Heinrich Heine University, Universitätsstr. 1, D-40225, Düsseldorf, Germany; Institute of Botany, Heinrich Heine University, Universitätsstr. 1, D-40225, Düsseldorf, Germany; Institute of Botany, Heinrich Heine University, Universitätsstr. 1, D-40225, Düsseldorf, Germany; Cluster of Excellence on Plant Science (CEPLAS), Heinrich Heine University, Universitätsstr. 1, D-40225, Düsseldorf, Germany

**Keywords:** pH electrode, iron, Arabidopsis, lookup tables (LUT), rhizosphere, root, pH, bHLH39

## Abstract

The rhizosphere, the region surrounding plant roots, plays a critical role in nutrient acquisition, root development, and plant–soil interactions. Spatial variations in rhizosphere pH along the root axis are shaped by environmental cues, nutrient availability, microbial activity, and root growth patterns. Precise detection and quantification of these pH changes are essential for understanding plant plasticity and nutrient efficiency. Here, we present a refined methodology integrating pH indicator bromocresol purple with a rapid, non-destructive electrode-based system to visualize and quantify pH variations along the root axis, enabling high-resolution and scalable monitoring of root-induced pH changes in the rhizosphere. Using this approach, we investigated the impact of iron (Fe) availability on rhizosphere pH dynamics in wild-type (WT) and bHLH39-overexpressing (39Ox) seedlings. bHLH39, a key basic helix–loop–helix transcription factor in Fe uptake, enhances Fe acquisition when overexpressed, often leading to Fe toxicity and reduced root growth under Fe-sufficient conditions. However, its role in root-mediated acidification remains unclear. Our findings reveal that 39Ox plants exhibit enhanced rhizosphere acidification, whereas WT roots display zone-specific pH responses depending on Fe availability. To refine pH measurements, we developed two complementary electrode-based methodologies: localized rhizosphere pH change for region-specific assessment and integrated rhizosphere pH change for net root system variation. These techniques improve resolution, accuracy, and efficiency in large-scale experiments, providing robust tools for investigating natural and genetic variations in rhizosphere pH regulation and their role in nutrient mobilization and ecological adaptation.

## Introduction

The rhizosphere, the soil zone influenced by plant roots, is crucial for many functions in plants [1]. This change in rhizosphere pH can occur due to the release of certain substances by the plant roots, such as organic acids, exudates, or protons [2]. The process can influence soil nutrient availability, microbial activity, and overall plant growth [3]. While it is well known that roots modify rhizosphere pH, the spatial and quantitative variations in these changes remain understudied. Understanding the mechanisms and regulation of rhizosphere pH is essential for developing strategies to improve plant nutrient uptake and enhance crop productivity.

Both rhizosphere acidification and alkalizations can be different on the different longitudinal section of the roots [4]. Bromocresol purple, a pH indicator, enables visual detection of pH changes within the range of 5.2–6.8 [5, 6]. However, distinguishing subtle differences between individual pH values or various root segments is challenging when relying solely on visual observation, as it does not provide accurate, quantitative pH values required for robust experimental analysis. To address this limitation, an electrode-based measurement system was employed to ensure precise and accurate pH readings, particularly in specific regions of interest along the root, referred to as localized rhizosphere pH change. Additionally, we developed an electrode-based system to quantify the net pH change induced by the rhizosphere of the entire root system, termed integrated rhizosphere pH change. These methodologies provide a robust and reliable approach for investigating root-associated pH dynamics, facilitating comparative studies across different environmental conditions and genetic variations.

Rhizosphere pH plays a crucial role in regulating the solubility and bioavailability of essential nutrients like iron (Fe), phosphate, and zinc and root–environment interactions. In this study, we investigated plant responses to varying levels of Fe availability, focusing on rhizosphere acidification and proton release. Apoplastic acidification act as a primary factor influencing rhizosphere pH dynamics. Root cells primarily acidify the apoplast by exporting protons, which then diffuse into the surrounding rhizosphere, maintaining an equilibrium between the apoplast and the rhizosphere. As a result, a proton gradient forms in the rhizosphere, while the apoplast remains a primary site of interest for Fe solubility. In most plant species other than grasses, Fe deficiency enhances proton release and rhizosphere acidification in the subapical region of roots [7–10]. A significant mechanism enhancing nutrient availability is rhizosphere acidification, facilitated by proton pumps such as Arabidopsis protein H^+^‐ATPase (AHA) located in the plasma membrane [11, 12]. This process lowers the pH of the surrounding soil, thereby simplifying nutrient uptake for the plant [13, 14].

This study aimed to develop precise methodologies for measuring rhizosphere pH changes induced by the entire root system as well as specific root regions, thereby highlighting differential root-zone effects. Specifically, we investigated the impact of Fe availability on rhizosphere pH dynamics in wild-type (WT) and bHLH39-overexpressing (39Ox) seedlings. bHLH39 is one of the transcription factor that is crucial for stimulating Fe uptake [15]. Overexpression of bHLH39 (39Ox) constitutively enhances Fe acquisition, leading to Fe toxicity. In our growth condition we see that iron toxicity leads to inhibition of the root length at the seedling stage. Consequently, 39Ox plants exhibit shorter root growth compared to WT at seedling stage, a response that is more pronounced in Fe-containing media than under Fe-deficient conditions [15]. Transcriptome analysis of 39Ox revealed that AHA7, S8H (Scopoletin 8-Hydroxylase), F6'H1 (Feruloyl-CoA 6ʹ-Hydroxylase1), PDR9 (Pleiotropic Drug Resistance 9) are among up-regulated in genes +Fe condition [15]. However, the effect of bHLH39 overexpression on root-mediated acidification remains unexplored. We hypothesized that this response is constitutively enhanced in 39Ox plants. The optimized protocol developed in this study facilitated precise characterization of genotype-specific rhizosphere pH variations, providing new insights into the role of bHLH39 in modulating root-associated pH changes.

## Materials and methods

**Figure 1.**
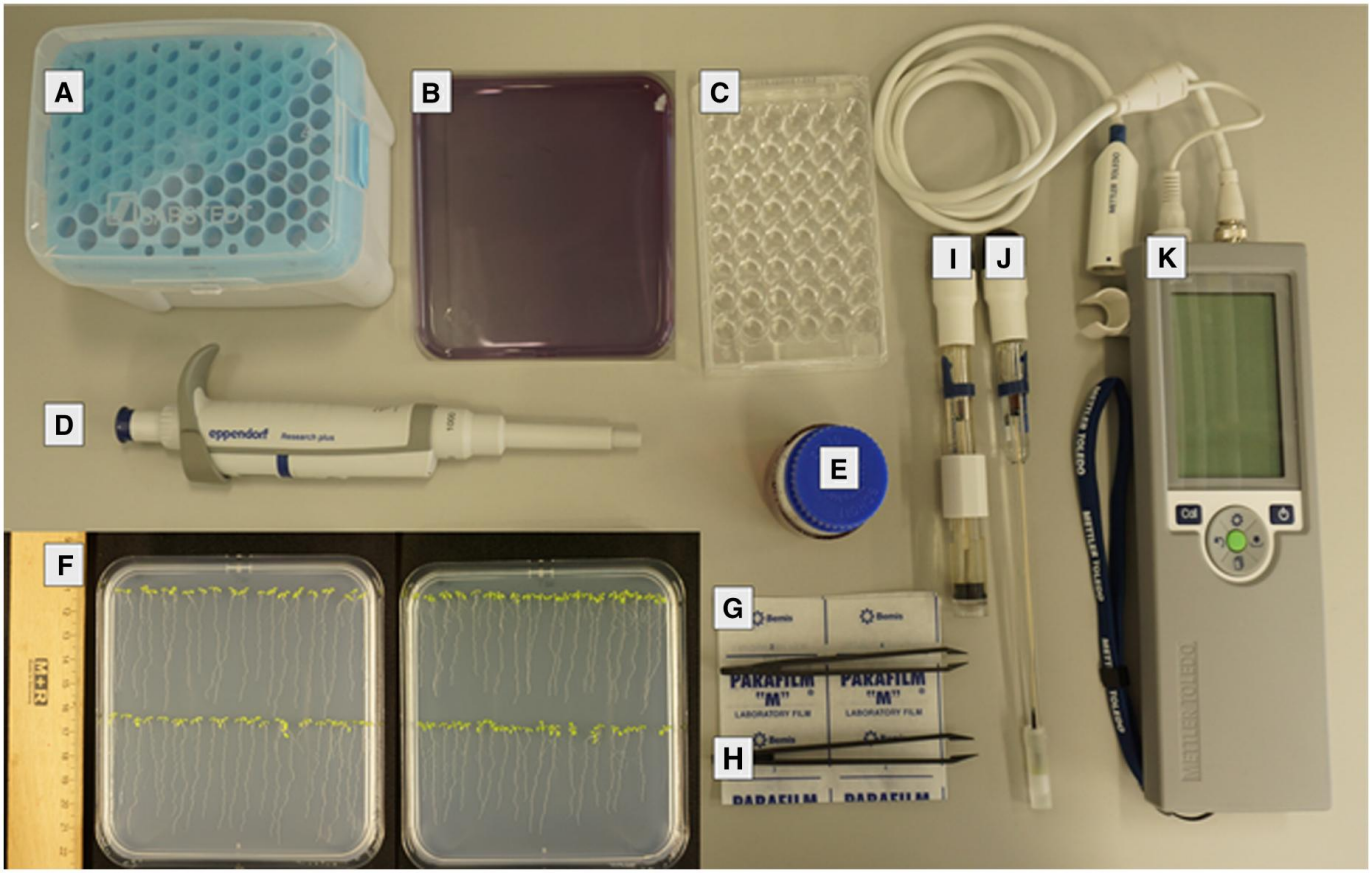
Required material and equipment. A: Pipette tips, B: Bromocresol purple reaction plate, C: 48-well plate, D: Pipette E: Bromocresol purple reaction solution, F: Seedlings in −Fe and +Fe condition, G: Parafilm, H: Plastic tweezers, I: Surface electrode, J: Electrode for liquid, K: pH measurement instrument

The required material and equipment for this study is shown in in the Fig. 1 and listed Supplementary list S1 and S2.

### Plant material

WT Arabidopsis ecotype Columbia-0 and bHLH039 overexpression (39Ox) lines [15] were used in this study.

### Plant growth media

Modified half-strength Hoagland agar medium was prepared as previously described in [16] (see Supplementary Data for details). The medium was supplemented with 50 μM Fe(III)NaEDTA for iron-sufficient conditions (+Fe), 0 μM Fe(III)NaEDTA for iron-deficient conditions (−Fe), and 150 μM Fe(III)NaEDTA for iron-excess conditions (++Fe).

### Plant growth conditions and treatments

Arabidopsis seeds were surface sterilized with sterilisation solution (6% sodium hypochlorite (NaOCl) and 0.1% TritonX) for 8 min and washed five times with distilled water. Seeds were stratified for 2 days in 0.1% plant agar in the dark at 4°C. Seeds were plated in two rows of 20 seeds on Hoagland agar medium containing Petri dishes. The plates were then placed vertically in a growth chamber with a temperature of 21°C and a 16-h light cycle from 6 am to 10 pm. The seeds were allowed to germinated, and vertically grown in square plates in different conditions for 10d under these conditions.

### Preparation of bromocresol purple reaction plate and solution

For bromocresol purple reaction plate, Add 0.006% (w/v) of bromocresol purple and 0.2 mM CaSO_4_ in sterile Milli-Q water. Adjust pH to 6.4 using KOH or HCl. Add 0.8% plant agar and heat in the microwave until completely dissolved. Pour the molten solution into Petri dishes (120 × 120 × 17 mm), dispensing 20–25 ml per dish. Ensure that the solution forms a uniformly thin layer. The solution is allowed to cool until solidified. For bromocresol purple reaction solution, add 0.006% (w/v) of bromocresol purple and 0.2 mM CaSO_4_ in sterile Milli-Q water. Adjust pH to 6.4 using KOH or HCl.

### Quantitative analysis of localized rhizosphere pH change by Arabidopsis root

**Figure 2.**
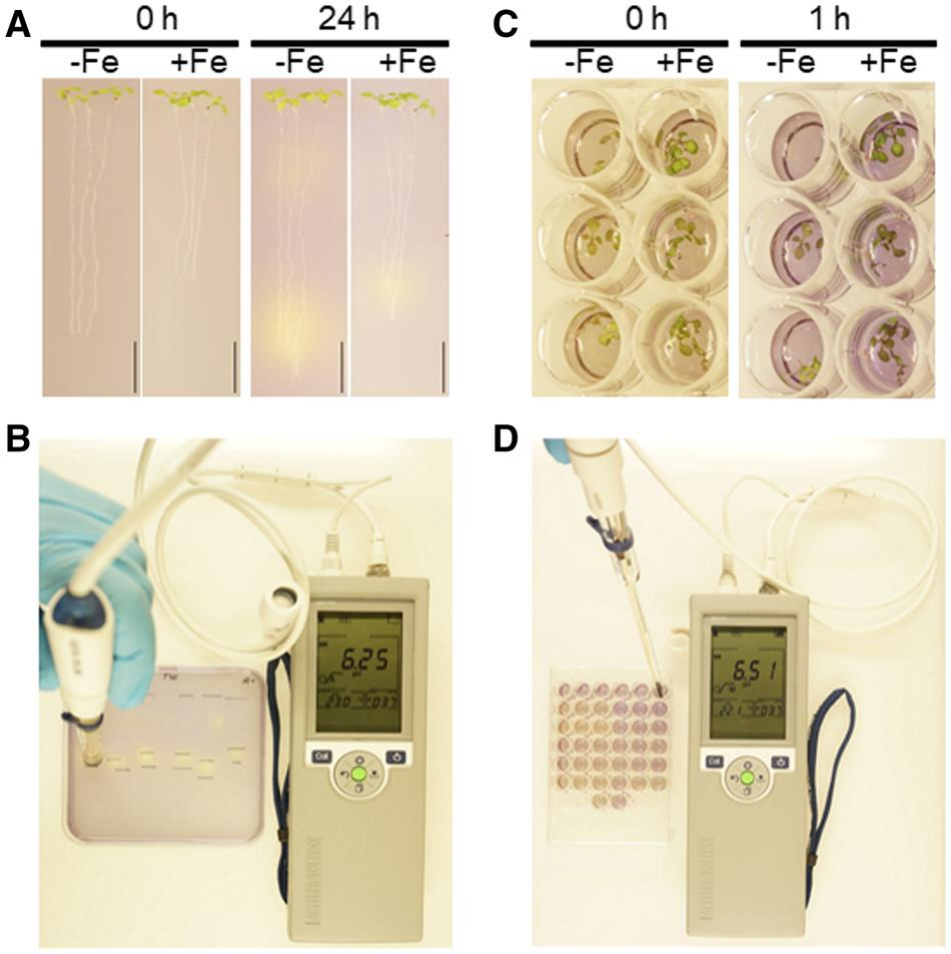
Representative images of procedure quantifying localized and integrated rhizosphere pH change in Arabidopsis. (**A**) Representative seedlings before and after incubation on Bromocresol purple reaction plate. The scale bar represents 1 cm. (**B**) Representative photograph of the procedure of the pH documentation after seedlings are removed for measurement of the pH after 24 h with surface pH electrode. Support lines were marked on the plate so that precise measurements could be taken after removing the seedlings. (**C**) Representative images of seedlings before and after incubation in bromocresol purple reaction solution. (**D**) Representative photograph of the procedure of the pH Measurement documentation after seedlings are removed after 1 h incubation in the bromocresol purple reaction solution

After being grown for 10 days under specific conditions using Hoagland media, the seedlings were gently transferred onto bromocresol purple pH indicator plates using sterilized tweezers. Three seedlings, with identical root lengths to ensure uniformity and to minimize interference in the acidification zone, were placed together on each plate. The plates were then returned to the growth chamber and incubated for 24 h (Fig. 2A). A control plate, without any seedlings, was included in the experiment. Following the incubation period, photographs of the plates were captured to document any pH-related changes. To analyze these changes, lookup table (LUT) images were generated for each plate using ImageJ software (https://imagej.nih.gov/ij/). A lookup table is a graphical representation that maps pixel intensity values to specific colors, enhancing the visualization of pH variations indicated by bromocresol purple. To accurately analyze pH changes in a targeted rhizosphere region, the seedlings must be carefully removed. To measure pH in specific regions, the pH Electrode InLab Surface Pro-ISM was employed. The pH of plates without seedlings was measured to determine whether pH changes occurred during incubation in the growth chamber (End pH; see Fig. 2B). An empty plate served as a control to facilitate consistent measurements throughout the experiment; which establishes a baseline (referred to as the Start pH) for subsequent comparisons of rhizosphere acidification intensity following seedling transfer. The change in the rhizosphere pH (ΔpH) on of bromocresol purple reaction plate was evaluated through the following steps:

ΔpH=End pH—Start pH

**Figure 3.**
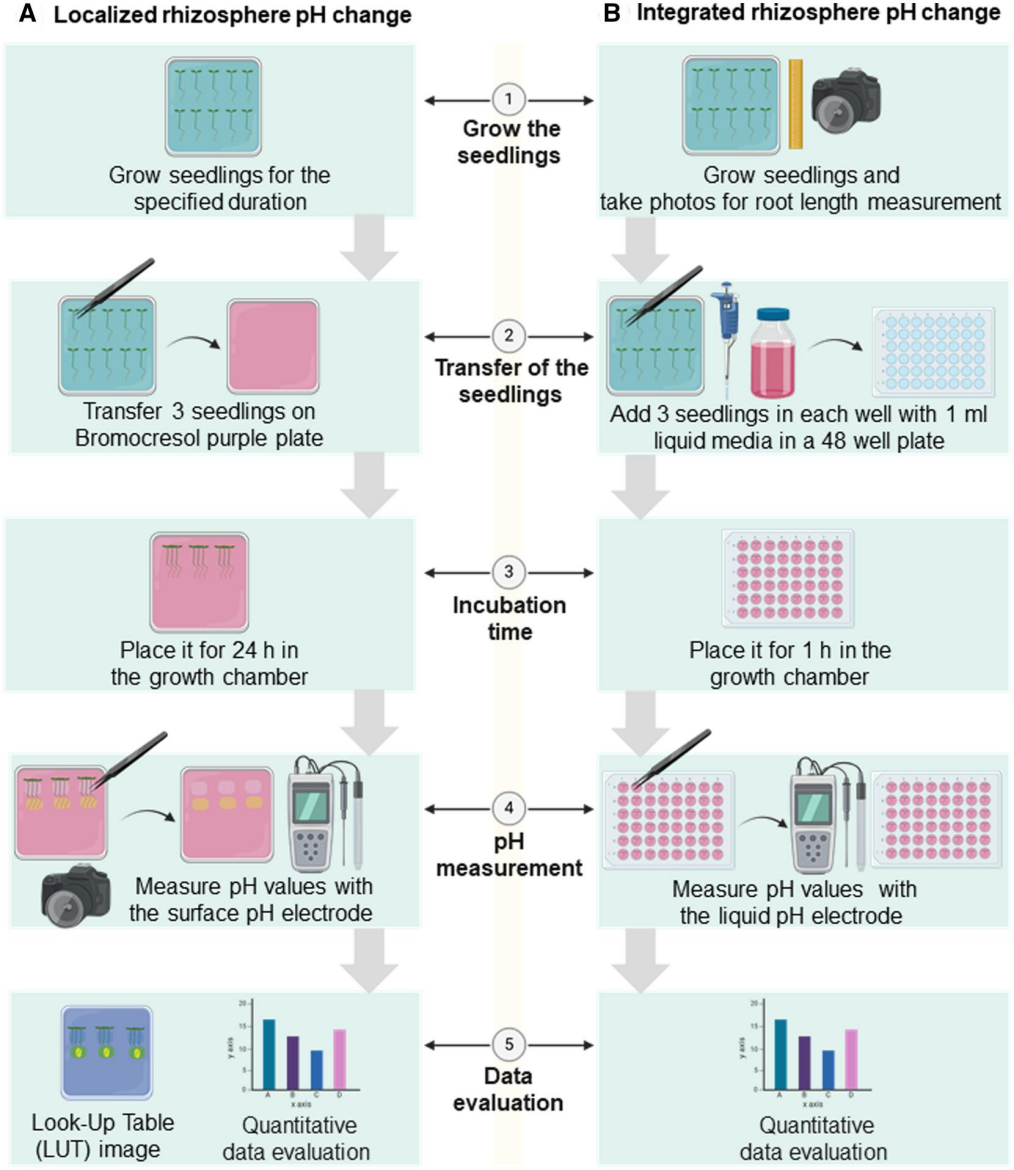
Graphic protocol scheme summary for quantifying localized and integrated rhizosphere pH change in Arabidopsis and the different steps involved. (**A**) Procedure for quantifying localized rhizosphere pH change. (1) Seedlings were grown for certain time points on growth media in the growth chamber; (2) transferred three seedlings together per replicate on bromocresol purple reaction plate; (3) incubate plate for 24 h in the growth chamber; (4) take the photograph of plate after 24 h and remove the seedling and take the pH reading of region of interest with surface pH electrode; (5) generate LUT image from photograph and analyze the data as mention in method section and creation of data graph. (**B**) Procedure for quantifying integrated rhizosphere pH change. (1) Seedlings were grown for certain time points on growth media in the growth chamber; take the photograph of plate and used it for calculating the sum of root length for three seedlings representative one replicate; (2) three seedlings together per replicate were transferred on 1 ml of bromocresol purple reaction solution in per well on 48 well plates; (3) incubate plate for 1 h in the growth chamber (4) remove the seedling and take the pH reading per well with liquid pH electrode (5) analyze the data as mention in method section and creation of data graph (C). Figure was prepared with BioRender

ΔpH was subsequently presented graphically. Negative values indicate a decrease in pH, positive values reflect an increase in pH, in the region under investigation. Graphic protocol is summarized in Fig. 3A.

### Quantitative analysis of integrated rhizosphere pH change by Arabidopsis root

To assess rhizosphere pH changes induced by whole roots, seedlings were grown for 10 days under specific conditions using Hoagland medium. Before the assay, photographs of the seedlings grown on agar plates were taken to measure root lengths (see section Root length measurement). For the assay, three seedlings were transferred to each well of a 48-well plate, ensuring that the roots were fully immersed in bromocresol purple reaction solution. The 48-well plate was then placed back in the growth chamber for 1 h (Fig. 2C). The pH changes were measured (referred to as End pH) using the pH Electrode InLab Micro Pro-ISM (Fig. 2D). An empty well served as a control and established a baseline (referred to as the Start pH) for subsequent comparisons of rhizosphere acidification intensity following seedling transfer. The sum of the root lengths of three seedlings per well was determined and the pH change determined per unit of root length (in cm) per time duration (in 1 h) in the given volume of the assay. Alternatively, root weights can be used. In our case, ΔpH × cm^−1^ × h^−1^ was calculated using the following formula:

$$\frac{\mathrm{End}\;\mathrm{pH}\;-\;\mathrm{Start}\;\mathrm{pH}}{\mathrm{Total}\;\mathrm{root}\;\mathrm{length}\;(\mathrm{in}\;\mathrm{cm})\;\times \;\mathrm{time}\;(1\;h)}\;$$

Negative values indicate a reduction in the rhizosphere pH, while positive values suggest an increase in the rhizosphere pH. The data were subsequently represented graphically to illustrate the relationship between root activity and pH changes. Graphic protocol is summarized in Fig. 3B.

### Root length measurement

WT and 39Ox plants were grown vertically in square plates for 10d. Plates were photographed. The photographs were used for the measurements of root lengths. Root length was measured from frontal images of plant roots using the JMicroVision software (http://www.jmicrovision.com) as described in [17].

### Statistical analysis

Three technical repeats and three biological repeats were used. Statistical data analysis was performed using one-way ANOVA followed by a Tukey’s test in OriginLab software. Graphs were created using OriginLab software. Different lowercase letters in bar graphs indicate statistically significant differences (*P* < .05).

## Results

### Quantifying localized rhizosphere pH change by Arabidopsis root

**Figure 4.**
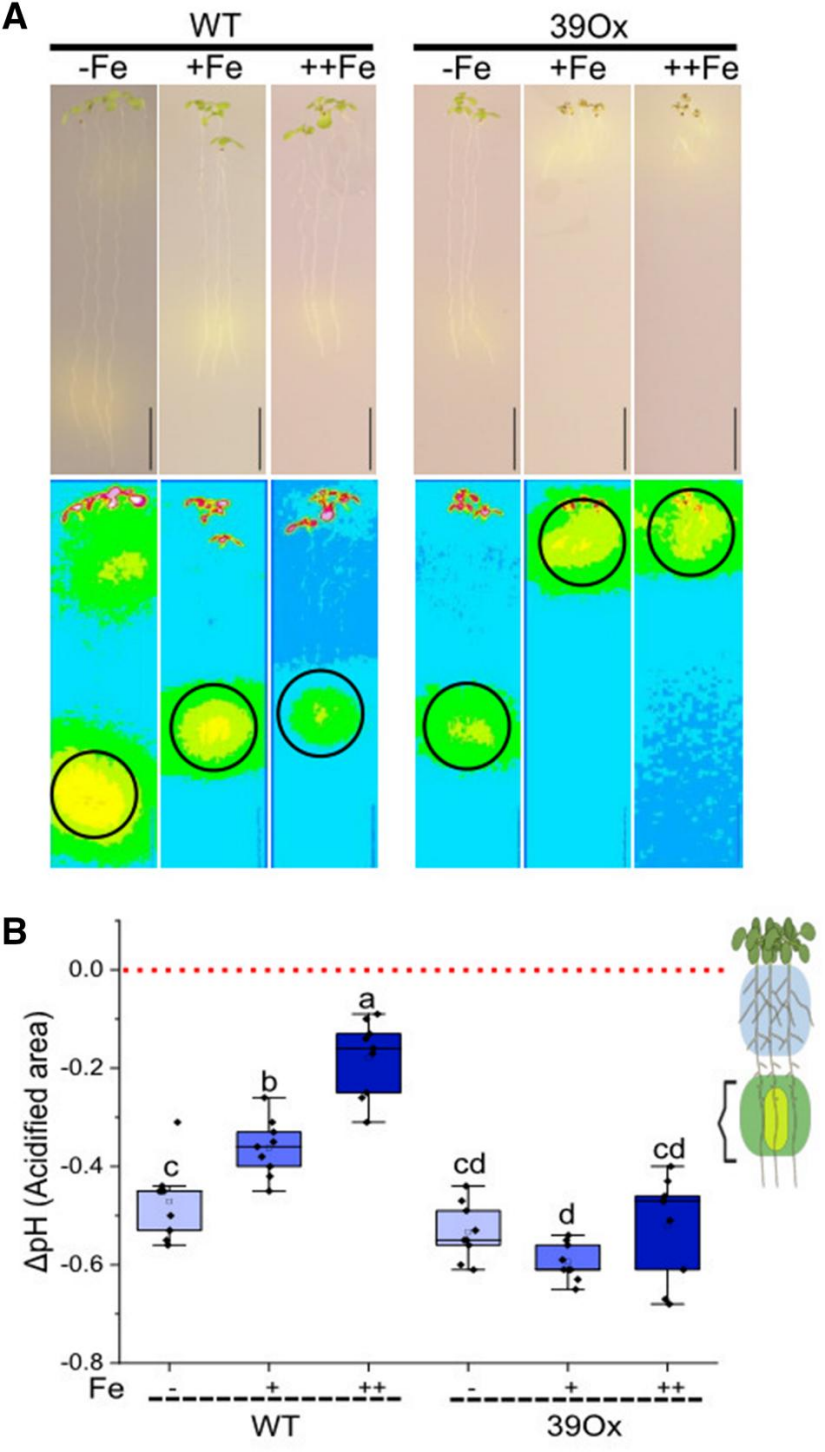
Quantifying the localized rhizosphere pH change in 10-day-old seedlings of 39Ox and WT under Fe sufficiency (+Fe), Fe deficiency (−Fe) and Fe excess (++Fe). (**A**) Rhizosphere pH change by Arabidopsis roots during 24 h on solid bromocresol purple media. One representative mages of seedlings per condition on solid bromocresol purple media are presented (Above). LUT images were used to intensify the visibility of the acidification (Below). Black circle represents the area where surface pH electrode was placed. (**B**) Change in rhizosphere pH by Arabidopsis roots after 24 h on solid bromocresol purple media at region of interest (the acidified area) by 10-day-old seedlings. Data presented as a bar diagram (*n* = 9, from 27 individual roots, one measurement per three seedlings). Letters indicating statistically significant differences; error bars are standard deviations (ANOVA and Tukey test, *P* < .05). Red line represents the no pH change axis on the graph

39Ox and WT seedlings were grown under Fe sufficiency (+Fe), Fe deficiency (−Fe) and Fe excess (++Fe) for 10 days. Subsequently, seedlings were transferred to bromocresol purple reaction plates for 24 h. Images were then captured to assess pH-dependent color changes, allowing for a comparative analysis of the two genotypes under different Fe conditions (Fig. 4A). The standard and LUT images of the seedlings clearly showed a decrease in the root lengths with increasing Fe concentration in 39Ox and WT seedlings (Fig. 4A). This root plasticity growth response was expected according to previous findings [16]. The LUT images clearly showed the differences between areas of acidification of the seedlings in different genotypes and conditions (Fig. 4A). In WT, affected rhizosphere acidification area decreased in response to Fe levels in the media. However, in the 39Ox plants, the affected areas remained relatively constant under the three Fe conditions examined (Fig. 4A).

The acidified area, visible as yellow, was used to measure pH at specific points. The graphs in Fig. 4B effectively illustrated pH variations in affected regions under different conditions. The red line represented as a neutral axis with no pH change; values above indicate basification, while values below indicate acidification. In WT, acidification of the affected area decreased with increasing Fe concentration. In contrast, the 39Ox line showed consistent acidification across all conditions (Fig. 4B). Notably, 39Ox exhibited similar acidification to WT under Fe-deficient conditions, but significant differences emerge under Fe-optimum and Fe-toxic conditions, where the 39Ox line showed higher rhizosphere acidification in every scenario.

### Quantifying integrated rhizosphere pH change by Arabidopsis root

**Figure 5.**
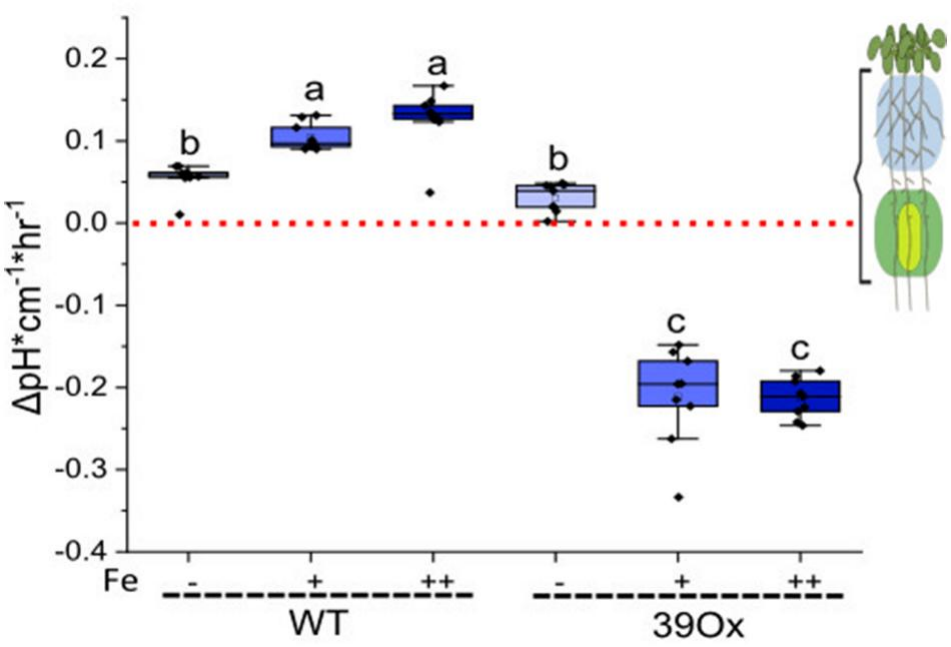
Quantifying the integrated rhizosphere pH change in 10-day-old seedlings of 39Ox and WT under Fe sufficiency (+Fe), Fe deficiency (−Fe) and Fe excess (++Fe). Rhizosphere pH change by whole Arabidopsis roots after 1 h on liquid bromocresol purple media. Data presented as a bar diagram (*n* = 9, from 27 individual roots, one measurement per three seedlings). Letters indicating statistically significant differences; error bars are standard deviations (ANOVA and Tukey’s test, *P* < .05). Red line represents the no pH change axis on the graph

The LUT images revealed differential pH changes in the rhizosphere along the entire root. To further evaluate the effect of the whole root on the surrounding media, electrode-based measurements were performed in bromocresol purple reaction solution. The graphs in Fig. 5 illustrated integrated pH variations introduced into the rhizosphere under different conditions. The red line represented as a neutral axis with no pH change; values above indicate basification, while values below indicate acidification. WT seedlings showed increased basification across all conditions, with higher Fe concentrations amplifying this effect. This implies that WT, basification was more prominent phenomenon than acidification. In contrast, the 39Ox line displayed variable responses: under Fe-deficient conditions, it exhibited minimal basification, while under Fe-optimum and Fe-toxic conditions, acidification significantly increased. Comparative analysis of the two genotypes revealed that the 39Ox line consistently showed increased acidification relative to WT across all conditions (Fig. 5). This underscores a genotype-specific response to Fe availability and highlights that different root zones have distinct effects on net rhizosphere pH.

## Discussion

Spatial pH variations along the root longitudinal axis have been shown to influence nutrient mobilization and root growth patterns [18]. This proton excretion is regulated by various genes and influenced by both developmental and environmental conditions [12, 19]. The low pH environment surrounding plant roots also influences root architecture, which is crucial for plant establishment [20]. Therefore, understanding the genetic control of rhizosphere acidification, particularly in a spatially resolved manner along the longitudinal root zonation, is essential for elucidating root development processes and enhancing nutrient acquisition, plant growth, and yield. To achieve precise and consistent differentiation of pH levels across the root system, raw image data was converted into a standardized color scheme using LUT. These LUT images facilitate quantitative analysis by providing a color-coded interpretation of pH distribution, allowing researchers to assess the acidification zones induced by root activity. The LUT, a color map that assigns specific colors to intensity values in the image, aids in interpreting pH changes on a bromocresol purple medium. Dye gives us the idea of the region of the differential pH variations in rhizosphere of root. The acidified regions, as indicated by the yellow color, were used to quantify pH changes in the rhizosphere. The quantification of pH changes in the acidification zones by the seedling’s roots ensured that pH differences were visually distinct and measurable, promoting more accurate and reproducible analysis of root-mediated acidification. The LUT imaging revealed genotype-specific differences in rhizosphere acidification, with the method enhancing qualitative and quantitative analysis of rhizosphere pH changes in specific regions. WT seedlings exhibited reduced root length and acidified area with increasing Fe concentration. In contrast, the 39Ox line, which overexpresses bHLH39, displayed relatively consistent acidification across all conditions. WT show highest acidification under Fe deficiency, which is a common response in plants to enhance Fe solubility and availability [21]. The quantification integrated rhizosphere pH of root give information about which process (acidification or basification) is prominent in the rhizosphere of root. Most interesting observation the entire root was acidified in the 39Ox in Fe sufficiency (+Fe), and Fe excess (++Fe) conditions. As therefore the integrated rhizosphere pH acidification of the solution only occurred in these conditions in 39Ox.

The method used in this study, which combines region-specific and whole-root pH measurements, was refined by selecting an appropriate number of seedlings per technical replicate to ensure reliable detection of pH variations. Three seedlings were used for this purpose to balance precision and experimental throughput. For imaging and LUT Analysis, several factors, such as lighting, manual camera settings, and image calibration, require standardization to improve the accuracy of pH quantification. To obtain more precise pH readings, optimization of root immersion time and volume was necessary. Regular calibration of pH electrodes was also critical to ensure consistency, particularly when detecting subtle pH changes under different Fe concentrations. This approach is not limited to the current study but can be adapted for application to other plant systems and various abiotic and biotic stress conditions.

However, for successful implementation under such circumstances, it is essential to optimize parameters such as exposure time, the number of plants per replicate, and the quantity of growth media. Using liquid growth media in various plant species could provide potential advantages for maintaining root integrity during transfer into the bromocresol purple reaction plate or solution. Also, studying rhizosphere pH changes across developmental stages can clarify its impact on growth, nutrient availability, root architecture, and stress responses.

In conclusion, rhizosphere pH changes are well-known to influence nutrient availability and root growth. The localized rhizosphere pH change method overcomes the limitations of conventional approaches, such as low resolution and limited quantitative accuracy, by providing a color-coded pH map and an electrode-based system for precise, region-specific measurements [6, 22, 23]. Additionally, the integrated rhizosphere pH change method offers a fast and efficient alternative to conventional spectrophotometric and visual techniques for assessing pH variations across genotypes and growth conditions [24, 25]. Integrating these complementary approaches enhances the interpretation of genotype- and environment-specific differences in rhizosphere pH regulation and nutrient mobilization. Overall, this study advances our ability to link rhizosphere acidification dynamics to root growth and nutrient availability, offering robust tools for investigating genetic and environmental regulation of rhizosphere processes. Rhizosphere pH plays a crucial role not only in regulating nutrient availability but also in influencing microbial activity and soil chemistry. In the future, these methodologies will be instrumental in understanding both natural and genetic variation in these traits, as well as its role in ecological adaptation.

## Supplementary Material

bpaf026_Supplementary_Data

## Data Availability

A full dataset for each method is available in Supplementary file S1. Raw image files are available upon request.

## Supplementary data

*Conflict*  *of interest statement*. None declared.
