# Supplementary material for: Quantitative tools for analyzing rhizosphere pH dynamics: localized and integrated approaches
Source: Biol Methods Protoc. 2025 Apr 3;10(1):bpaf026. doi: 10.1093/biomethods/bpaf026 (PMC12036966; doi:10.1093/biomethods/bpaf026)
Supplement: bpaf026_Supplementary_Data [file bpaf026_supplementary_data.zip › bpaf026_Supplementary_Data.zip!/Supplementary data.docx]

**Supplementary data**

**Supplementary list S1.** List of materials and equipment used in this study.

1. Growth chamber to grow plant material (Percival scientific, model: CU-36L4)
2. Autoclave (Systec, model : D-45)
3. pH measurement instrument (METTLER TOLEDO, model: Seven2Go^TM^, catalogue number: 30207949)
4. Surface pH Electrode (METTLER TOLEDO, model: InLab Surface Pro-ISM, catalogue number: 30249570)
5. Liquid pH Elektrode (METTLER TOLEDO, model: InLab Micro Pro-ISM, catalogue number: 51344163)
6. Digital camera (Sony Alpha 6000)
7. Square petri dish 120x120x17 mm (Greiner Bio-one, catalogue number: 688161)
8. 48-well plates (VWR® Tissue culture plate, non-treated and sterilised, catalogue number:736-0349)
9. Pipette (Eppendorf Research® plus, volume 100-1000 µl, catalogue number 3123000063)
10. 1000-µl tips (Sarstedt, catalogue number: 70.3050.020)
11. Bemis™ Curwood Parafilm™ (catalogue number :10018130)
12. Plastic tweezers
13. Microwave

**Supplementary list S2.** List of chemicals used in this study.

A. Seed sterilisation
   1. 6% sodium hypochlorite (NaOCl) and 0.1% TritonX
   2. Milli-Q water
B. Hoagland media
   1. Macronutrient (concentration)
      a. MgSO_4_ · 7H_2_O (0,75 mM)
      b. KH_2_PO_4_ (0,5 mM)
      c. KNO_3_ (1,25 mM)
      d. Ca (NO_3_)_2_ · 4H_2_O (1,5 mM)
   2. Micronutrient (concentration)
      a. KCl (50 µM)
      b. H_3_BO_3_ (50 µM)
      c. MnSO_4_ · H_2_O (10 µM)
      d. ZnSO_4_ · 7H_2_O (2 µM)
      e. CuSO_4_ · 5H_2_O (1,5 µM)
      f. (NH_4_)_6_Mo_7_O_24_ · 4H_2_O (0,075 µM)
   3. Iron: FeNaEDTA
   4. Sucrose (1%)
   5. Plant agar (1.4%)
   6. Set pH 5.8
C. Bromocresol purple reaction plate and solution
   1. Milli-Q water
   2. 0.006% Bromocresol purple
   3. 0.2 mM CaSO_4_ · 2H_2_O
   4. 0.8 % plant agar (without for Bromocresol purple solution)
   5. Set pH 6.4
